# Antibacterial Activity and Chemical Analyses of the Alkaloidal Fraction of *Neltuma laevigata* (Humb. & Bonpl. Ex Willd) Britton & Rose Inflorescences

**DOI:** 10.3390/molecules30244714

**Published:** 2025-12-09

**Authors:** Uriel Nava-Solis, Mario Rodriguez-Canales, Ana Bertha Hernandez-Hernandez, Cesar M. Flores-Ortíz, Marco Aurelio Rodriguez-Monroy, Maria Margarita Canales-Martinez

**Affiliations:** 1 Laboratorio de Farmacognosia, Unidad de Biotecnología y Prototipos, Facultad de Estudios Superiores Iztacala, Universidad Nacional Autónoma de México, Tlalnepantla de Baz 54090, Mexico; biol.navasolis@gmail.com (U.N.-S.); mario.rodcan09@gmail.com (M.R.-C.); ana.b.hdez@iztacala.unam.mx (A.B.H.-H.); 2 Laboratorio de Fisiología Vegetal, Unidad de Biotecnología y Prototipos Laboratorio Nacional en Salud, Facultad de Estudios Superiores Iztacala, Universidad Nacional Autónoma de México, Tlalnepantla de Baz 54090, Mexico; cmflores@unam.mx; 3 Laboratorio Nacional en Salud, Facultad de Estudios Superiores Iztacala, Universidad Nacional Autónoma de México, Tlalnepantla de Baz 54090, Mexico; 4 Laboratorio de Investigación Biomédica en Productos Naturales, Carrera de Medicina, Facultad de Estudios Superiores Iztacala, Universidad Nacional Autónoma de México, Tlalnepantla de Baz 54090, Mexico; dr.marcorodriguezmonroy@gmail.com

**Keywords:** piperidinic alkaloids, mesquite, antibacterial activity, julifloridine

## Abstract

Species of the *Neltuma* syn *Prosopis* genus are known for their use in traditional medicine in America, Asia and Africa. The use of the leaves, bark and inflorescences of one species widely distributed in the arid zones of Mexico, *Neltuma laevigata* (Humb. & Bonpl. Ex Willd) Britton & Rose, has been reported for the treatment of ocular, gastric and skin infections. Its activities have been related to different secondary metabolites, particularly phenylpropanoids and alkaloids. In the present study, the antibacterial activity of the alkaloidal fraction of inflorescences of *P. laevigata* collected in Zapotitlán Salinas, Puebla, México, against *Staphylococcus aureus* ATCC 25,923 and *Vibrio cholerae* CDBB-1159 was studied by Kirby–Baüer and broth microdilution tests, and its activity on plasmatic membranes was later identified using a protein leakage assay and fluorescence microscopy. Subsequently, the alkaloidal fraction was separated via chromatographic methods, and the purified compounds were elucidated using nuclear NMR and HRESIMS analysis. The alkaloidal fraction showed an important antibacterial activity, with a possible effect on the cytoplasmic membrane of the tested strains. Julifloridine, a piperidine alkaloid previously reported in the genus, was identified for the first time in this species.

## 1. Introduction

The emergence of resistant pathogens (bacteria, fungi, parasites) currently represents one of the main challenges to public health at the global level [1]. This phenomenon occurs when viruses, bacteria, fungi, and parasites do not respond to antimicrobial treatments, thus allowing the survival of the microorganism within the host [2]. This problem has been addressed through different lines of research, with a main one being the investigation of secondary metabolites, in which either new alternatives or complements are sought to recover the antimicrobial activity of already known drugs [3].

One of the main problems with secondary metabolites is their availability, which depends mostly on environmental stress, the organ that produces them and the age of the organism [4,5]. However, a good guide for these studies is ethnopharmacological research, that is, the compilation of data obtained from research into different forms of traditional medicine, especially herbal medicine, in which not only the use of plants for the treatment of various diseases has been studied, but also the organ(s) most conducive to the healing effect and the quantity required [5,6].

A plant genus of importance in traditional medicine in various arid regions of the Americas is *Neltuma*, whose members are popularly known as “mesquites,” “algarrobos,” or “acacias” [7,8]. Its use by communities living in arid zones is widely documented, highlighting its importance as timber, agricultural material, food, and a medicinal plant [8]. An important example is *Neltuma laevigata* (syn *Prosopis laevigata*), a species widely distributed in Mexico, which has recently been studied due to reports of its potential use against diseases related to bacterial infections [7,8,9].

There are records of the use of decoctions of their roots and aerial organs (mostly bark, branches, leaves and flowers) in traditional medicine for the treatment of diseases of microbial origin, especially skin, oral, and eye infections [9,10,11]. Studies on the therapeutic potential of those species report that a significant portion of this activity is associated with the nitrogenous secondary metabolites present in the various organs studied, mainly phenylpropanoids, like phenolic acids, coumarins, and flavonoids, and piperine and pyrrolizidine alkaloids [7,8,9,10,11].

Of the alkaloids, the piperidine type has been reported to have important biological properties, including neurotoxic and antimicrobial properties, usually attributed to their capacity to interact with the plasmatic membrane and nucleic acids [12,13,14,15,16]. Some studies [17,18,19] show that piperidine alkaloids likely exhibit activity on the plasmatic membrane of diverse pathogens, such as bacteria, yeasts (mainly *Candida* strains), phytopathogen fungi, and diverse parasites. Accordingly, the main objective of this research was to determine the antibacterial activity of the alkaloid extract of *P. laevigata* inflorescences, characterizing its extracts and identifying their possible effect on cellular membrane integrity.

## 2. Results

### 2.1. Alkaloidal Extract Obtention

The inflorescences of *N. laevigata* were collected in Zapotitlán Salinas municipality, at 18°19′20.6″ N, 97°31′26.8″ W. A total of 164.9 g of dried material was obtained.

The alkaloidal extract was prepared using the acid-basic method. The obtained extract was named AZS (alkaloids of Inflorescences from Zapotitlán Salinas), and a total of 1032.7 mg was obtained, corresponding to 0.626% of the total dried material used. The presence of alkaloids in the extract was confirmed by thin-layer chromatography using Dragendorff’s reagent.

### 2.2. Antimicrobial Assays

#### 2.2.1. Bacterial Inhibition and Determination of MIC

The results of bacterial inhibition and MIC determination are shown in Table 1. It is noted that the minimal inhibitory and bactericidal concentrations over *V. cholerae* CDBB-1159 are lower than those obtained over *S. aureus* ATCC 25923, but higher than those for chloramphenicol, used as a reference compound.

#### 2.2.2. Effect on Bacterial Cell Membrane Integrity

##### Protein Leakage Assay

**Figure 1:**
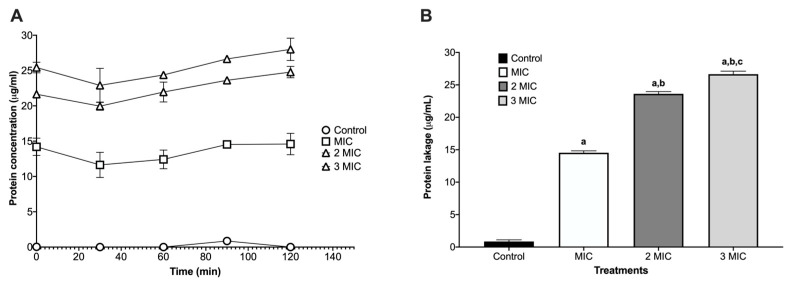
Protein leakage kinetics on *Staphylococcus aureus* ATCC 25923. (**A**) Time-course of protein released into the supernatant (µg/mL) over 120 min exposure. The protein released into the supernatant (µg/mL) was quantified after exposure to MIC, 2MIC, and 3MIC. (**B**) Endpoint at 120 min displayed as bars (mean ± SD, *n* = 3). Leakage increased with the increase from MIC to 3MIC, consistent with progressive membrane damage. Letters above the bars denote significant differences in multiple comparisons (one-way ANOVA, *p* < 0.0001): a = different vs. control; b = different vs. MIC; c = different vs. 2 × MIC.

The protein leakage assay on *Staphylococcus aureus* ATCC 25923 revealed dose-dependent membrane damage after 120 min of exposure. The control showed minimal release of proteins (µg/mL), consistent with intact membranes. At the MIC, extracellular protein increased, indicating initial permeabilization of the lipid bilayer. Leakage rose stepwise at 2MIC and 3MIC, peaking at 3MIC, which is consistent with progressive disruption of the cell envelope and loss of permeability control (Figure 1).

##### Identification of Bacterial Cell Membrane Damage by Fluorescence Microscopy

**Figure 2:**
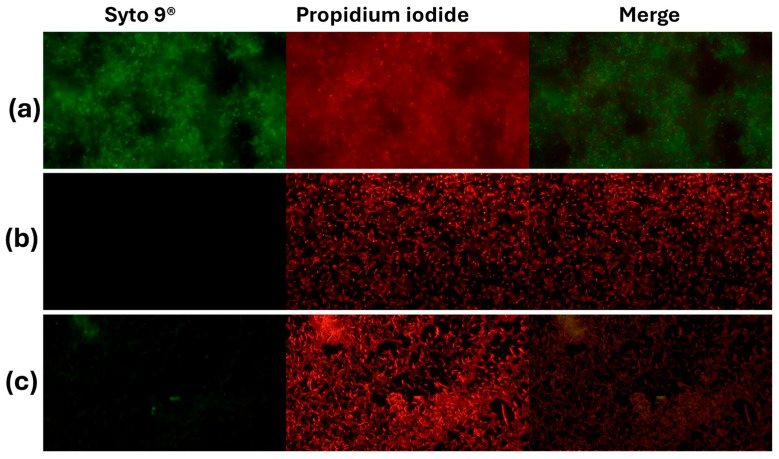
Activity of the alkaloidal extract on the membrane of *S. aureus* ATCC 25923. The bacteria were stained with Syto 9^®^ and propidium iodide and visualized by inverted microscopy at 400× total magnification. Syto 9^®^ and propidium iodide were viewed at wavelengths of 483 nm and 636 nm, respectively. (**a**) Control (NaCl 0.85%), (**b**) positive control (70% isopropanol); (**c**) AZS extract (9.7 µg/mL).

**Figure 3:**
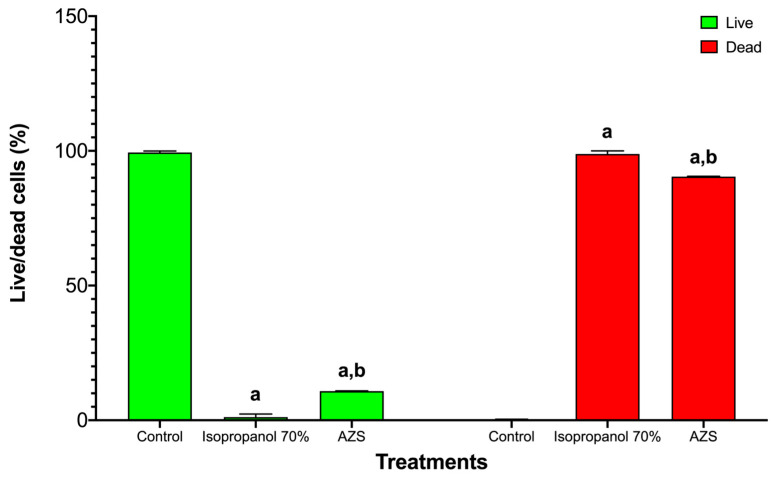
*S. aureus* ATCC 25923 viability was reduced by 70% isopropanol and AZS. Bars are grouped by viability state: live (green) on the left and dead (red) on the right. Within each state, bars correspond to control, 70% isopropanol, and AZS (mean ± SD, *n* = 3). Two-way ANOVA showed significant main effects and a significant interaction (*p* < 0.0001), indicating that both 70% isopropanol and AZS reduced the live fraction and increased the dead fraction vs. control. Letters denote pairwise differences within each viability state: a = different vs. control; b = different vs. 70% isopropanol.

In *S. aureus* ATCC 25923, the live/dead viability assay showed the expected sharp dose/effect-oriented split between live (green) and dead (red) populations across treatments. The untreated control consisted almost entirely of live bacteria with negligible death, indicating intact membranes and metabolic activity (Figure 2). Exposure to 70% isopropanol resulted in near-complete killing, with the live fraction collapsing to the background and the dead fraction dominating—consistent with rapid solvent-mediated membrane disruption. AZS markedly reduced viability, yielding a low live fraction and a high dead fraction that was similar to the results observed under isopropanol conditions and indicating a bactericidal rather than merely bacteriostatic effect under these conditions (Figure 3).

**Figure 4:**
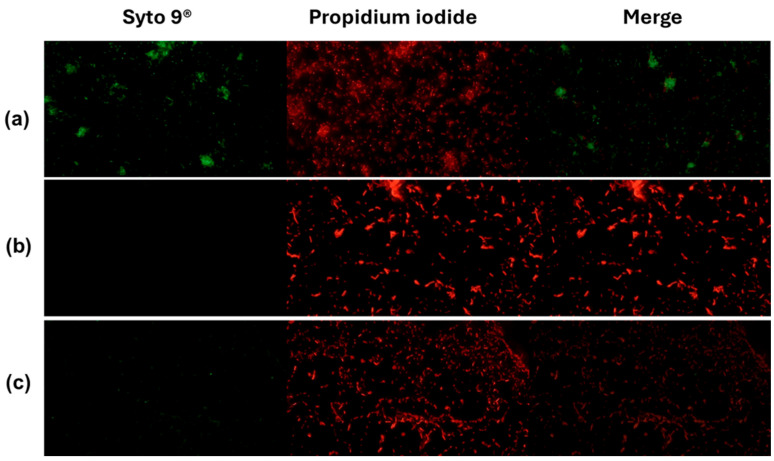
Activity of the alkaloidal extract on the membrane of *V. cholerae* CDBB-1159. The bacteria were stained with Syto 9^®^ and propidium iodide and visualized by inverted microscopy at 400× total magnification. Syto 9^®^ and propidium iodide were viewed at wavelengths of 483 nm and 636 nm, respectively. (**a**): Control (NaCl 0.85%); (**b**): positive control (70% isopropanol); (**c**): AZS extract (4.9 µg/mL).

**Figure 5:**
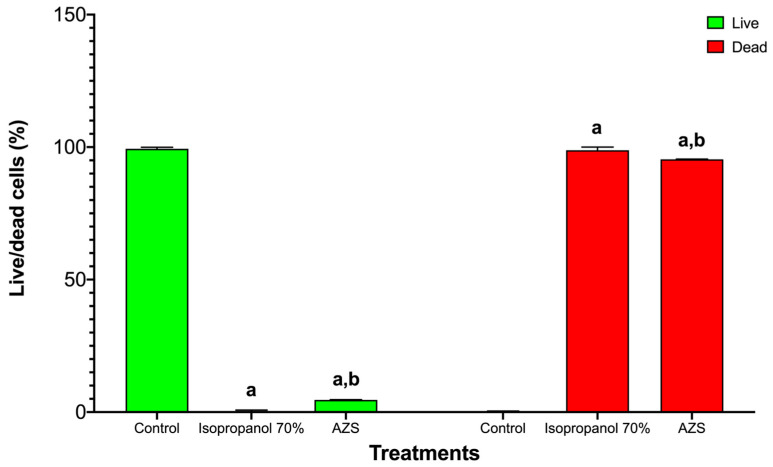
Loss of viability in *Vibrio cholerae* CDBB-1159 after exposure to 70% isopropanol or AZS. Bars are arranged by viability state (live, green; dead, red). Within each state, treatments are control, 70% isopropanol, and AZS; values are mean ± SD (*n* = 3). Two-way ANOVA detected significant main effects and an interaction (*p* < 0.0001), showing that both treatments reduced the live fraction and increased the dead fraction relative to the control. Letter code: a = different from control; b = different from 70% isopropanol (comparisons performed within each viability state). Comparison between the treatments shown significative differences in the damaged cell percentages between the isopropanol and AZS extracts (*S. aureus* ATCC 25923 t = 13.18, *p* = 4.30; *V. cholerae* CDBB-1159, t = 4.96, *p* = 4.30).

In *Vibrio cholerae* CDBB-1159, the live/dead assay showed a marked loss of viability upon treatment. Controls were predominantly live with negligible death, indicating intact membranes. Isopropanol (70%) collapsed the live fraction to near background and yielded a dead fraction close to the maximum, consistent with fast solvent-mediated membrane disruption. AZS also reduced viability, with a small residual live fraction and a large dead fraction, supporting a bactericidal effect under these conditions (Figure 4 and Figure 5).

### 2.3. Chemical Identification of Alkaloids

**Figure 6:**
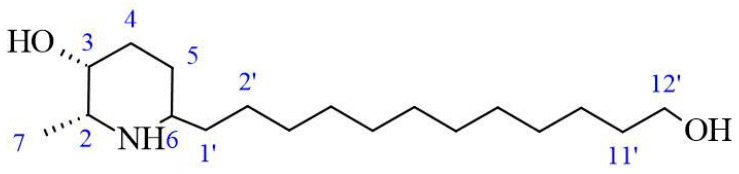
Structure of julifloridine.

**Figure 7:**
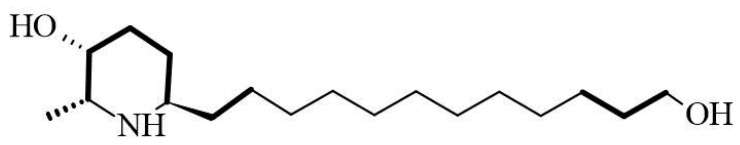
COSY correlation of julifloridine.

**Figure 8:**
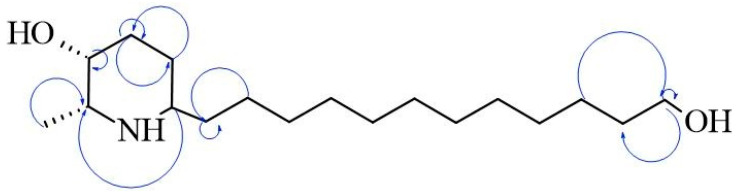
Key HMBC correlations of julifloridine.

The AZS extract was fractionated via flash chromatography over Si-gel columns. The presence of alkaloids was confirmed in four of 12 fractions, and the most abundant fraction was selected for purification by chromatographic methods (see the Section 4). From this, as the alkaloid julifloridine was identified as one of the main components in the alkaloid extract (Table 2). Its structure was determined by HRESIMS (*m*/*z* 300.2895 [M + H]) and ^13^C and ^1^H NMR experiments. The results of these experiments are shown in Table 2 and Figure 6, Figure 7 and Figure 8. Two-dimensional NMR experiments helped to corroborate this structure: In COSY experiment (Figure 7), the correlations between δH 3.20 (q, *J* = 6.6 Hz, H-2) and δH 1.52 (m, H-7) and δ3.81 (dt, *J* = 3.4, 1.7 Hz, H-3) and δH 1.78 (qt, *J* = 8.7, 3.2 Hz, H-5a) were found and confirmed the structure of the piperidine ring. The bond between the piperidine ring and the aliphatic chain was confirmed by the correlation between δH 3.02 (tt, *J* = 8.7, 4.2, H-6), δH 1.64 (dt, *J* = 5.3, 15 Hz, H-1′a) and δH 1.28 (m, H-2′). A consistent correlation between all protons at positions 2′ to 9′ was observed due to the characteristic multiplet of the signals. Finally, the terminal hydroxyl (at the 12′ position) was verified due to the correlations between δH 3.51 (t, *J* = 6.7 Hz, H-12′) and δH 1.51 (m, H-11′) and δH 1.31 (m, H-10′). Based on the HMBC experiment (Figure 8), the position of all groups in the alkaloid was confirmed: the correlation between δH 1.52 (m, H-7) and δC 56.15 (C-2), established the position of the methyl substituent, and between δH 3.51 (t, *J* = 6.7 Hz, H-12′) and δC 32.31 (11′) and δC 25.6 (C-10′), confirming the presence of the hydroxyl substituent at position 12′.

## 3. Discussion

The antimicrobial activity of different extracts from aerial parts of plants in the *Neltuma* genus is widely recognized in traditional medicine in different parts of the world. The biological activity of different organ extracts has hence been studied.

Different studies [20,21] have analyzed the correlation between antimicrobial activity and piperidine alkaloids. In the specific case of *Neltuma laevigata*, most studies on its antimicrobial activity are based on organic extracts, and some of these studies attribute its activity to the presence of alkaloids, mainly piperidine alkaloids [10,22,23,24].

The total percentage of the alkaloidal fraction in *N. laevigata* inflorescences, 0.2%, is higher than other values reported in different aerial parts of *Neltuma* species like *N. alpataco*, *N. argentina*, *N. flexuosa* and *N. pugionata* [25]. The concentration is similar to that reported in *Neltuma africana* in 2009 [26] but lower than that reported in *P. chilensis* [25] and different extracts from *N. juliflora* aerial parts, including methanolic, ethanolic and aqueous extracts [27,28]. In other studies with *N. laevigata,* the total alkaloid concentration (1032.7 mg AE/g of dry biomass) is higher than that reported previously by Nava-Solis et al. (11.87 mg AE/g of dry biomass) [10] and Herappe-Mejía et al. (612.64 mg AE/g of dry biomass) [29]. Both studies were performed using methanolic and ethanolic extraction. These kinds of extraction could affect the total alkaloids extracted, since more efficient extraction of this class of secondary metabolites generally requires the use of techniques based on pH changes, in addition to the use of solvents of intermediate polarity such as chloroform and dichloromethane, depending on characteristics like the structure and nitrogen core [30,31]. Other explanations for the different alkaloidal concentrations could be the metabolic differences between *Neltuma* species and the use of different organs, as the distribution and accumulation of alkaloids (and other secondary metabolites) depend on the age and activity of the plant organ [32]. For the flower specifically, the concentration of secondary metabolites is altered in response to biological need, for example, to attract pollinators, deter herbivores and defend against microbial infections. Thus, the accumulation of toxic compounds, such as alkaloids, in flowers could be crucial for the survival of this organ [33].

The results of antimicrobial tests (Table 1) are comparable with other studies with different species of *Neltuma* species. For the activity on *S. aureus* ATCC 25923, the MIC is less than that obtained by Raghavendra et al., 2009 [13], and Nava-Solis et al., 2022 [10], with different organic solvent extracts. Regarding alkaloid extracts, Singh et al. reported a higher MIC (100 µg/mL) than the present study in an alkaloidal extract of *N. juliflora* flowers [17], and in 2013, dos Santos et al. reported an MIC of 50 µg/mL on *S. aureus* in a basic chloroformic pod extract [14]. In this study, we compared the MIC value obtained for the AZS extract (9.7 µg/mL) with those of several known antimicrobial agents. AZS exhibited a higher inhibitory concentration than linezolid (2 µg/mL), vancomycin (in a range of 1–2 µg/mL) dalbavancin (in a range of 0.032–0.1 µg/mL), telavancin (0.094–0.125 µg/mL) and melittin (8 µg/mL), and daptomycin (0.03–5 µg/mL); was comparable to the reported MIC-ranges of penicillin (0.03–64) µg/mL; and was lower than those reported for colistin (>64 µg/mL); and oxacillin (0.064–256 µg/mL) [34,35,36]. In the case of *V. cholerae* CBBB-1159, Napar et al. [37] reported higher MIC values for methanolic extracts of *N. cineraria* and *N. juliflora* than the present study (1 mg/mL), and Taheri et al. reported an MIC of 2 mg/mL for a hydroalcoholic bark extract of *Neltuma* sp. [38]. Other studies using organic extracts only found a susceptibility effect, observed using the Kirby–Baüer assay [10,39,40], showing a medium–high inhibition activity depending on the concentration, organ and species. We compared the MIC value obtained for the AZS extract (4.9 µg/mL) with previously reported MICs of antibiotics tested against different *V. cholerae* strains. The AZS extract showed a higher MIC than those reported for tetracycline (0.5 µg/mL), ciprofloxacin (0.0005–0.002 µg/mL), norfloxacin (0.004–1 µg/mL) and azithromycin (0.19–2 µg/mL); comparable MIC values to those reported for furazolidone (0.5–16 µg/mL), trimethoprim (4–128 µg/mL) gentamicin (1–16 µg/mL) and sulphamethoxazole (1–16 µg/mL); and lower MIC than ampicillin (32–128 µg/mL) [41,42]. Although most known antibiotic compounds exhibit lower MIC values than AZS extract, it is important to consider that, in an organic extract, a substantial proportion of constituents may exert antagonistic effects on overall antimicrobial activity [43]. Julifloridine, the main alkaloid identified in this study, has been reported previously in the *Neltuma genus* [22,44] as part of the group of piperidine alkaloids ubiquitously distributed in all alkaloid-rich plant parts [45]. It has also been reported previously as the main alkaloid in *N. juliflora* flowers [17,22,46]. Identification and structure elucidation were carried out using nuclear magnetic resonance and mass spectrometry experiments. The signals obtained in 1H and 13C NMR are similar to those previously reported [17,22], and the mass obtained in mass spectrometry (299.28076) has been previously reported in *N. juliflora* inflorescences [22]. Regarding its antibacterial properties, Singh and Verma, in 2011, reported this alkaloid as responsible for antibacterial activity in alkaloidal extracts from some aerial organs of *Neltuma juliflora* [17]. However, julifloridine has an amphipathic structure (Figure 5, Figure 6 and Figure 7), a characteristic that allows it to affect cell membrane integrity [23,47,48,49]. Other alkaloids with similar structures have been reported to have important effects on different cell walls or cytoplasmatic membranes of other biological groups. Prosopine, for example, has been reported to have inhibitory activity over *Fusarium solani*, and this activity was related to its interaction with the cell wall and cytoplasmatic membrane, which could affect the enzymatic activity of Beta-glicosidase [50]. (−) spectaline and (−) cassine were reported to be antileishmanial agents with a probable activity against enzyme arginase, an important virulence factor for *Leishamania amazonensis* [51].

Although there are few reports on the activity of piperidine alkaloids of the genus *Neltuma* on the bacterial cell plasma membrane, studies on *Neltuma* species have identified several bioactive alkaloids whose antibacterial activity has been mainly associated with membrane disruption, increased permeability, and interference with cellular metabolism or bioenergetic pathways. Julifloricine, an alkaloid from *N*. *juliflora*, has been associated with increased membrane permeability and leakage of intracellular contents [51]; In the case of juliprosine and juliprosopine, they have been linked to cell membrane disruption, collapse of membrane functions [14]. Other alkaloids reported in *N. juliflora*, *N. glandulosa*, mainly prosopine, juliprosopine, juliflorine, prosoflorine, and cassine are the most common, with a probable activity against phospholipidic membranes [18,21,45,46,47,48,49]. These activities have been associated with the structural features of these alkaloids, highlighting their nitrogenous, lipophilic cyclic structure and their amphipathic constitution, which have a potential insertion between the phospholipids in the bacterial cell membrane [23,47,48,49]. On the other hand, activity against the plasmatic membrane has been documented previously, mainly in organic and aqueous extracts of leaves and pods of *N. juliflora* on different bacterial strains. Importantly, activity by protein leakage in *S aureus* has been observed [28]. The alkaloidal fractions of the *Neltuma* genus have different activities in different eucaryotic cells (including erythrocytes [24,50] and glial cells [51]), microorganisms and cellular organelles like mitochondria In the present study, protein leakage and fluorescence microscopy experiments confirmed the activity against the plasmatic membrane. In the protein leakage experiment, the activity of the AZS extract and the concentration of proteins in the extracellular medium remained constant throughout the experiments, and a possible dose–response effect was observed, as significant differences were found between the three treatments groups (F = 3388.53, *p* = 4.07) (Figure 1). The fluorescence microscopy assay confirmed the activity of the alkaloidal fraction against the cell membrane of the analyzed strains, as all bacterial cells were completely dyed with propidium iodide, which can only cross cell membranes that have alterations in their structure and/or permeability; this effect can be seen in both the treatments and the positive control, isopropanol. This result confirms the activity of the alkaloidal extract on the cytoplasmatic membrane. This effect has also been previously reported in floral methanolic extracts [10] and in an aqueous *Neltuma cineraria* bark extract [52]. These results may also confirm the reports of piperidine alkaloid activity in cells, which highlights their potential to intercalate between phospholipids [21,53,54,55,56,57,58] and could be related to processes such as disruption of membrane integrity, destabilization of ion gradients and possible interference with metabolic pathways. Nevertheless, identifying the specific membrane-related mechanisms of Julifloridine requires further targeted studies to confirm its precise mode of action.

## 4. Materials and Methods

### 4.1. Extract Obtention

Fresh inflorescences of *Neltuma laevigata* (syn. *Prosopis laevigata*) were collected in March 2022 in Zapotitlan, Puebla, Mexico. The plant material was taxonomically identified by Patricia Jáquez Ríos (M.Sc.). Voucher specimens were deposited in the IZTA herbarium (IZTA 3223). Samples were dried at room temperature and ground to a powder, yielding 164.95 g of material.

The total amount of dried *N. laevigata* inflorescences was defatted using 100 mL of reagent-grade hexane. The sample was acidified with 100 mL of water adjusted to pH 2 with a 1 N HCl stirred continuously for 6 h and then filtered. The filtrate was transferred to a separation funnel and partitioned with 100 mL of reagent-grade chloroform. This extraction was performed three times, and the organic phases were discarded.

The acidic aqueous phase was then alkalinized with 100 mL of water previously adjusted to pH 10 with 1 N NaOH solution, followed by further partitioning with 100 mL of reagent-grade chloroform. After basification, the pH of the mixture was measured at 7.5. The combined organic phases were recovered and concentrated under reduced pressure using a rotary evaporator IKA Rv 10 auto V (IKA-Werke GmbH & Co, Staufen, Germany). The presence of alkaloids in the final extract was confirmed using Dragendorff’s reagent (Darmstadt, Hesse Germany).

### 4.2. Antibacterial Assays

*Bacterial strains*

*Staphylococcus aureus* ATCC 25923 and *Vibrio cholerae* CDBB-1159 were used.

#### 4.2.1. Antibacterial Activity Assay

Antibacterial activity was evaluated using the Kirby–Baüer agar diffusion test, with Müeller–Hinton agar as the culture medium. Five-millimeter-diameter disks (Whatman no. 5, Little Chalfont, Buckinhamshire, UK) were impregnated with 2 mg of each alkaloidal extract, and disks with 25 µg of chloramphenicol were used as positive controls, by triplicate. The cultures used were adjusted to a concentration of 1 × 10^8^ CFU/mL using an optical density 0.5 McFarland standard probe (620 nm) on a Thermo plate reader, model Multiskan EX, type No. 355, serial No. 3550902720 (Waltman, MA, USA). After 24 h, the inhibition area diameter was measured, and the average of the measurements was calculated. The results were analyzed using one-way ANOVA [59].

#### 4.2.2. Determination of Minimal Inhibitory Concentration (MIC)

To evaluate the MIC, a broth microdilution assay was performed using sterile 96-well microplates (Corning, NY, USA). Müeller–Hinton broth was used as the culture medium and a serial concentration range of 1000–0.48 µg/mL per alkaloidal extract. The cultures were adjusted to a concentration of 1 × 10^5^ CFU/mL using an optical density McFarland standard probe (620 nm) on a Thermo plate reader, model Multiskan EX, type No. 355, serial No. 3550902720 (Waltham, MA, USA) [54]. The results were obtained by triplicate, adding 50 µL of a solution of triphenyl tetrazolium chloride (TTC) (0.08%) dissolved in sterile distilled water, as an indicator to visualize bacterial growth [60].

#### 4.2.3. Evaluation of Plasmatic Membrane Damage

##### Quantification of Protein Leakage

To evaluate the activity of the alkaloidal extracts on the cellular membrane, bacterial protein leakage was quantified using the protein leakage technique with *S. aureus* ATCC 25923 cultures at a concentration of 1 × 10^6^ FCU and each alkaloidal extract (at the MIC) by triplicate, having as control cultures without extract. Samples of 100 µL were taken every 30 min in a period of 2 h and the extracellular medium concentration was quantified via a Bradford assay. The absorbance at 595 nm was determined using a Thermo plate reader, model Multiskan EX, type No. 355, serial No. 3550902720 (Waltman, MA, USA). The results were expressed in micrograms of albumin equivalent per milliliter (µg e BSA/mL) and were analyzed by one-way ANOVA analysis [61].

##### Fluorescence Microscopy

*S. aureus* cultures at a concentration of 1 × 10^6^ were centrifuged in Eppendorf^®^ tubes at 10,000× *g* for 15 min in a Hentich D-78532 microcentrifuge (Andreas Hettich GmbH & Co., Föhrenstr, Tuttligen, Germany) to concentrate and remove the culture medium. The resulting culture was washed with a sterile physiologic solution (NaCl 0.85%) three times. Afterwards, the bacterial pellets were dissolved in alkaloidal extract solutions (1/2MIC, MIC and 2MIC), in isopropanol as positive control, and in saline solution (0.85%) as a control, for 30 min. Then, the cultures were centrifuged at 10,000× *g* for 10 min and washed with physiological solution. The supernatant was then removed and the pellets were suspended in 50 µL of a solution of Syto 9^®^ 6µM and propidium iodide (30 µM) for 15 min. Finally, 5 µL samples were taken and observed using an Oxio Inverso OX.2453—PLF inverted fluorescence microscope (Euromex Microscopen B.V., Duiven, The Netherlands) with the EX 540 fluorescence filter (BP25: 528–552 DM565 (LP) EM 620 (BP06 590–650 nm)) for propidium iodide and the EX480 filter (BP40: 460–500 nm DM510(LP) EM535 (BP50 660–660 nm)) for Syto 9 ™ (Thermo Fisher Scientific, Waltham, MA, USA) by triplicate. The images were analyzed with Image J 1.54k software (National Institute of Health (NIH), Bethesda, MD, USA) and the results were analyzed by one-way ANOVA using Graphpad Prism 9.4.1 software.

### 4.3. Purification and Elucidation of Alkaloidal Extracts

#### 4.3.1. Separation by Flash Chromatography

The AZS extract (800 mg) was adsorbed on Celite 545 (Thermo Fischer Scientific, Waltman, MA, USA) and fractioned via flash chromatography using a CombiFlash RF Lumen system (Teledyne Technologies Inc., Thousand Oaks, CA, USA) equipped with a photodiode array (PDA), an evaporative light-scattering detector (ELSD), and a 24 g RediSep *R*_f_ Gold-Sigel column (20–40 µm spherical, 60 Å, Teledyne Technologies Inc.) Samples were eluted using a gradient between *n*-hexane-CHC_3_-CH_3_OH with a flow rate of 12 mL/min: ELSD parameters were set as follows: spray temperature of 30 °C and a drift temperature of 60 °C. the PDA wavelengths were screened from 200 to 400 nm. Twelve fractions were obtained, and fractions AZS-8 to AZS-12 were positive for alkaloid presence in Dragendorff’s reactive test. The majority fraction, AZS-9 (100 mg), was used in subsequent purification.

#### 4.3.2. Fraction Purification by Column Chromatography (CC)

Fraction AZS-9 was adsorbed on Silica gel (Thermo Fischer Scientific, Waltman, MA, USA) subjected to CC using a glass column of 30 mm diameter × 500 mm length Silica gel (100 g) (Macherey-Nagel GmbH & Co., Düren, Germany) was used as the stationary phase and elution was performed with CHCl_3_-CH_3_OH, (100:0 → 0:100); each mobile phase was alkalinized with diethylamine 3%. A total of 135 aliquots were obtained and pooled in 12 fractions based on their TLC profile; the presence of alkaloids in the fractions was verified with Dragendorff’s reagent (Darmstadt, Hesse Germany). Fraction AZS-9.28 was the majority (14 mg) and was used in subsequent chemical analyses.

#### 4.3.3. Fraction Analysis by HRESIMS

Data were acquired using a Q Exactive Plus system (Thermo Fisher Scientific Waltman, MA, USA) equipped with an electrospray ionization source with an HCD cell. Data were collected in both positive and negative modes via direct injection or through an Acquity UPLC system (Waters Corporation, Milford, MA, USA). A BEH C18 column (50 × 2.1 mm i.d., 1.7 μm; Waters Corp. Milford, MA, USA) was used with the following gradient solvent system: from 15:85 to 100:0 CH3CN-0.1% aqueous formic acid for 10 min at a flow rate of 0.3 mL/min.

#### 4.3.4. Compound Purification via HPLC

Analytical and semipreparative HPLC was carried out on a Waters HPLC system equipped with a 2535 quaternary pump, a 2707 autosampler, and 2998 PDA and 2424 ELSDs using Gemini C18 and Kinetex C18 columns (5 μm, 110 Å, 250 × 4.6 mm i.d. and 5 μm, 110 Å, 250 × 10 mm i.d.; Phenomenex, Torrance, CA, USA) for analytical and preparative runs, respectively. Data were acquired and managed with Empower 3 software (Waters Corp. Milford, MA, USA) Fraction AZS-918 was solved in MeOH:dioxane 1:1 and purified using a gradient solvent system 15:85 to 100:0 CH_3_CN-H_2_O and a flow rate of 24.21 mL/min. The peak collection period lasted 15 min, starting at minute 6 and ending at minute 21, during which 7 mL were collected in 10 mL test tubes. The fractions were subsequently combined according to their UPLC-MS profiles (as describing below) affording six compounds, with the mayor constituent being compound AZS-918.1 (2.1 mg).

#### 4.3.5. Compound Analysis via UPLC-MS

The purified compounds were analyzed by ultra-performance liquid chromatography tandem mass-spectrometry (UPLC-MS), using a Waters Aquity UPLC-H Class (Waters, Milford, MA, USA) equipped with a quaternary pump, sample manager, column oven and photodiode array detector (PDA), coupled to a SQD2-quadrupole mass spectrometer with an electrospray ionization (ESI) source. The analysis was performed using a reverse-phase C18 column (Waters BEH C18 column 1.7 μm; 50 × 2.1 mm) and a gradient solvent system from 20:80 to 100:0 CH_3_CN-H_2_O (0.1% formic acid) at a flow rate of 0.3 mL/min. PDA detector was set from 190 to 500 nm and the mass spectrometer parameters were the following: cone and capillary voltage, 35.0 V and 3.0 kV, respectively; source and desolvation temperature, 350 and 450 °C, respectively; collision gas, N2; and mass range, *m*/*z* 50–2000 (scan duration of 0.5 s). All samples were analyzed in both positive and negative modes.

#### 4.3.6. Elucidation of Compounds by NMR Experiments

^1^H (600 MHz) and ^13^C (151 MHz) NMR, as well as 2D, HSQC, COSY and HMBC experiments, was performed in Me-OD d_4_ a JEOL ECS-400 (JEOL Ltd., Tokyo, Japan) spectrometer equipped with a high-sensitivity JEOL Royal probe. A total of 2.1 mg of the compound was dissolved in 200 µL of Me-OD d_4_. Data were processed using MestReNova 15 software (Mestrelab Research, S.L., Santiago de Compostela, Spain).

## Figures and Tables

**Table 1:** Inhibitory activity (diameter inhibition zone) and MIC of alkaloidal extract.

Strain	AZS	
	Diameter Inhibition (mm) *	MIC (µg/mL) *
*S. aureus* ATCC 25923	20.67 ± 1.15	9.7
*V. cholerae* CDBB-1159	20.33 ± 0.58	4.9
Control (Chloramphenicol)	19.33 ± 0.58	1.9

* Values are expressed as the mean of three independent replicates. Standard deviations are provided for the inhibitory activity assay. In the MIC assay, standard deviation could not be calculated because no variation was observed among replication measurements.

**Table 2:** ^1^H (600 MHz) and ^13^C (151 MHz) data for Julifloridine in Me-OD d_4_ (δ in ppm, *J* in Hz).

Position	Julifloridine	
	*δ*_H_	*δ*_C_
2	3.20, q, *J* = 6.6 Hz	56.1
3	3.81, dt, *J* = 3.4, 1.7 Hz	64.5
4a	1.93, dt, *J* = 10, 3.2 Hz	29.7
4b	1.64, dt, *J* = 5.3, 15 Hz	29.7
5a	1.78, qt, *J* = 8.7, 3.2 Hz	22.3
5b	167, m	22.3
6	3.02, tt, *J* = 8.7, 4.2	57.4
7	1.52, m	14.6
1′a	1.64, dt, *J* = 5.3, 15 Hz	33.4
1′b	1.42, dd, *J*= 5.8, 11.3 Hz	33.4
2′	1.28, m	24.9
3′	1.28, m	29.4
4′	1.28, m	29.4
5′	1.28, m	29.3
6′	1.28, m	29.3
7′	1.28, m	29.3
8′	1.28, m	29.3
9′	1.28, m	29.1
10′	1.31, m	25.6
11′	1.51, m	32.3
12′	3.51, t, *J* = 6.7 Hz	61.7

## Data Availability

The original contributions presented in this study are included in the article. Further inquiries can be directed to the corresponding author.
